# Extracts and Gleanings

**Published:** 1876

**Authors:** 


### Extracts and Gleanings.

Dr. A. G. Smythe, of Baldwyn, Miss., has been using salicylic acid in a number of cases of diptheria with the most happy effects. He uses the article first as a local application to the diseased part in any stage he finds it, either by applying it in the dry acid, by blowing it out of a quill, or by dipping a damp camels'-hair pencil or moss into it, and applying to the affected parts; or by mixing in thin syrup-of-squills, and use every four or five hours as a gargle. He also gives from one to four grains, according to the age of the child, in a sufficient quantity of glycerine; alternating at from three to four hours with from two to six drops of the tinct. chlor. iron, in a teaspoonful of syrup, other incidental indications to be met upon general principles. He has succeeded, so far, to his entire satisfaction in all cases when the treatment had been adopted before they were in a dying condition.

A Worm Passes Through the Wall of the Abdomen—Recovery.—*By W. R. Hurst, M. D.*—A little girl, daughter of Mr. C——, of DeKalb county, fell from a wagon upon a rock, which produced a severe contusion just below the short ribs on the right side, about three inches from the umbilicus. She suffered considerably for a time, but in a few hours revived and went to play; yet continued to complain, from time to time, of pain in the bowels. Constipation ensued, and it was necessary to give laxatives daily. The urine became scant and highly-colored, and the child became languid and dull.

On January 1st, about one month after the accident, I was called, and found her with considerable febrile excitement and symptoms of acute pentonites. There was a large lump on the right side of the abdomen, which was exceedingly tender.

A full dose of calomel was given, and the bowels ordered to be kept open with laxatives. Apprehending an abcess, I endeavored to promote resolution. After several days, finding I could not dissipate the swelling, I began the use of poultices, to encourage the formation of pus, and hasten its progress to the surface; in the meantime keeping an eye to the condition of the bowels and urine, using nitrous ether and watermelon seed tea as diuretics.

Soon the tumor pointed, and was opened. A copious discharge of pus ensued, and continued discharging, though declining in quantity, until February 2d, when the child complaining of a tickling sensation, the mother examined the place, and found three or four inches of a worm protruding from the abcess. She drew forth a lumbricoid about six or eight inches in length. Since then the place closed rapidly, and now (February 10th) no discharge appears; no pain is felt, and the child is fast recovering her health. At no time was any fecal matter discharged from the orifice; nor did there seem to be any serious obstruction to the evacuation of the bowels.

[The above case is certainly remarkable, and is important as teaching the possibility of recovery in what might be supposed as necessarily fatal injury to the bowels. In this case we opine that a rupture of the intestine occurred—probably the ascending colon—adhesive inflammation set up, and the bowel became attached to the inner wall of the abdomen, and an abcess formed, through which the worm made its exit.—Ed.]

Treatment of Scarlatinal Albuminuria.—Dr. Vesey, in the *Irish Hospital Gazette*, gives a case treated by turpentine and vinegar, from which is the following extract:

The anasarca was very much increased all over the body. The urine had been almost totally suppressed. During the previous thirty-six hours not more than four ounces (if so much) had been passed. This was of the color of tawny port wine. The immediate treatment has a hot bath, with mustard, followed by hot stupes to loins, a brisk purgative, and a turpentine enema. Turpentine confection was also administered in fifteen grain doses, every hour, and venegar and water (1 to 4) was given, *ad lib.*, as a drink. The bowels acted freely, and in three hours from the commencement of the treatment there was an improvement; the convulsions were not so severe, nor so frequent. Chloroform was also tried, but I did not derive the benefit therefrom that I expected, so did not persevere in its use.

In twelve hours the convulsions ceased, and did not return. The turpentine confection was now given every third hour, and did not produce any strangury. The quantity of water was notably increased—six ounces in twelve hours. He drank freely of vinegar and water, and was much pleased with it. He had very copious sweating, which continued for several hours.

Dec. 23.— To-day patient much better; pale and weak, but otherwise well; plenty of urine secreted, only a trace of albumen; *no blood or casts* could be found. From this date the convalescence was uninterrupted and complete.

I need not enlarge on the condition of the kidneys in this case. It will be sufficient to say that it was regarded as a case of masked scarlatina in the first instance, with the usual renal sequelæ, from exposure to cold. This view is borne out of the appearance of scarlatina in a sister of this boy a few days afterwards.

The reasons for the employment of turpentine are too obvious to be commented on. The vinegar was given with the idea of making the urea-poisoned blood purge itself of the offending matter through the skin. I do not venture to say that the diaphoresis was *propter hoc*, though certainly it was *post hoc*.

In the current number of *St. Bartholomew's Hospital Reports* will be found a very valuable paper by Dr. Reginald Southey, who prescribed sulphurous acid and compound spirit of horseradish in acute Bright's disease. Of vinegar he says, "I do not attribute her improvement very greatly to the large amount of *vinegar* in horseradish sauce that this patient took; and oftentimes since, in the persistent sickness of the uræmic state, I have given the dilute acetic acid of the pharmacopœ, in drachm or half drachm doses, with almost invariable benefit."

The following article on the use of gallic acid in albuminuria is by Dr. J. T. Jameson, in the *American Practitioner*: "I wish," he says, "to call attention to the use of gallic acid in the treatment of albuminuria as a sequel to scarlet fever, with which, in a few cases, I have met with marked success. My experience with the remedy has been as follows:

" In my first case, occurring in a boy aged about twelve years, the symptoms were very severe. There was œdema of the face and lower extremities, but no effusion into the thoracic or abdominal cavities; violent headache; blindness; there had been four or five strong epiliptiform convulsions; urine was scanty and contained blood, resembling exactly water in which fresh beef had been washed, and coagulating about one-half on testing with heat and nitric acic. To relieve the cerebral symptoms, a blister was applied to the neck, sinapisms to the extremities and lumbar region, cold to the head, and two or three doses of a mercurial with bitartrate of potassa. This was followed by iodide of potassium and a teaspoonful of a saturated solution of gallic acid every two hours. The acid was given in this manner for five days and nights in succession, the patient rapidly improving under its use, and the urine becoming more copious and less bloody. It was continued for twenty-two days, only at longer intervals, and at that date the urine when tested manifested the slightest possible trace of albumen, although the boy at this time was around the house and apparantly perfectly recovered, having been so for a number of days. The tinct. ferrischloridi was given in small doses, and completed the cure.

" My second case occurred in a girl about six years of age. The eruption was very livid and the skin had desquamated. The child recovered well from the fever, and was about the house. She went into a cold room to play with other children, and a day or two afterwards the face became œdematous; there was pain in the head; slight fever; urine quite bloody, and on testing in the usual manner presented considerable coagulation. The patient was put upon a saturated solution of gallic acid, a teaspoonful every two hours. In seven days the urine was free from albumen and copious in quantity, and the child seemed well, with the exception of debility, for which the muriated tincture of iron was prescribed. About ten days after this, in consequence of fresh exposure to cold, there was a slight relapse, the urine becoming again bloody and the face puffed; but on resuming the gallic acid for a few days these symptoms speedily subsided and the recovery became permanent. In this case the gallic acid was administered unaccompanied by any other medicine, except an occasional dose of castor oil to regulate the action of the bowels."

"*Remarks.*—The treatment hitherto generally adopted in this affection has been that of acting derivatively on the bowels by means of mercurials, followed by such diuretics as digitalis, sweet spirits of nitre, acetate of potash, etc.; but if future experiments should confirm the efficiency of gallic acid, I cannot but think we shall possess a remedy superior to any of the above. The gallic acid, if I understand its action aright, enters the blood unchanged, and unchanged is carried directly to the congested and inflamed capillaries of the secernent portion of the kidneys, acting as an astringent and tonic upon them, promoting their contraction, and thus arresting the exudation of red blood corpuscles and promoting the normal secretion of urine. I have seen no unpleasant effects from its administration as freely as above represented. It does not disturb the stomach, nor interfere with the appetite or digestion, but it does tend to constipate the bowels somewhat, rendering necessary the occasional use of a laxative."

MASTURBATION IN CHILDREN.—Dr. Jacobi says: "A boy or girl at play on the floor; they usually prefer the hard floor, and sometimes will select it in preference to a soft cushion or mattress. All at once they get quiet, cross the thighs, look a little pale in the face, soon become flushed, start up, remain a little immovable, eyes start now and then; they become flushed and excited, and afterwards exhausted. Such attacks have been reported to him as occurring several times during the day. A number of cases have been reported to him where the children were closely watched, and seen playing with the genital organs, especially boys while in bed; and not only play with the penis, but finally erect it, and at last succeed in squeezing a small amount of liquid from the orifice of the urethra; and that this occurred just at the time the face became flushed and the eyes started in their sockets. The results of this habit in children are about the same as in the adult, as far as the languor and general irritability of the nervous system are concerned. Such children are irritable, frequently a little bloated; have that peculiar shyness which is present in adults when adicted to the habit; have a peculiarly sallow skin, and the sebaceous follicles are exceedingly well developed.

"The sebaceous follicles in the skin of the infant are very large, and when about one year old they get smaller, but when this habit is contracted these follicles remain enlarged especially about the face. Probably a great many of the comedones seen upon the face of adults are the result of masturbation. Not all examples of this condition are due to this cause, but whenever such a face is seen it reminds one of masturbation, and probably in many instances they are due to theinfluence of this habit. In boys affected by such habits erection of the penis. especially in the beginning, is a very common symptom. Now and then a case will be found which may be recognized by a simple inspection of the penis. When the habit has been continued for some time the glans become enlarged, looks irritated, is reddened, and the prepuce is swolen. It has been stated that the same condition is sometimes seen about the genitals of girls, perhaps more frequently than in boys, and that there is a free secretion from the Bartholine glands and muciparous follicles about the orifice of the urethra. The labia majora also will be very large, and the clitoris is almost invaribly enlarged. It is said that there is a peculiar redness of the passages which can be easily diagnosticated from the redness of the mucous membrane of the vagina, which is so frequently seen in the cases of leucorrhoea occurring in small children.

"Bromide of potassium is serviceable in preventing children from masturbating during the intervals of sleep, and also to procure sleep. Alkalies may be used to the extent of relieving what little catarrh of the bladder may be present. To diminish the general irritability of the parts, lupuline and camphor are of service. Strychnia will be beneficial in many cases, as it is a generally useful remedy in all forms of nervous disease caused by masturbation, either general or local in character: and marked either by symptoms of partial paralysis or symptoms of irritation.

"Hypochondria, when it occurs in children, was believed always to be due to masturbation. Epilepsy and chosea have been produced by the habit. Local symptoms of debility are essentially those of more or less localized paralysis. Symptoms of loca lirritation are much more frequent. A peculiar *hysterical cough* such as heard in hysterical females, is occasionally heard among boys and girls, and in a large number of cases can be traced to masturbation as the cause. The doctor then related two cases, both occurring in *boys*, in whom the hysterical cough was a prominent spmptom. *Hemicrania* was mentioned as a disease not infrequently found in children 8 or 9 years of age, as well developed as in the adult, and produced by self-abuse. Iron and balladonna are serviceable in the treatment of this symptom.

"Another symptom found occasionally in children 7 years of age is neuralgia about the joints. This is much more frequent than has been observed in former years. The knee joint is very frequently involved, especially the internal condyle of the femur; also the styloid process of the ulna, and very frequently the vetebral column. Excessive masturbation is the cause not infreqently in these cases.

"Dr. Leaming mentioned incontinuance of urine as a symptom in children given to masturbation. In some of these cases the application to caustic to the prepuce and clitoris, just sufficient to make them a little tender, had been attended with much benefit. In the asylum with which he is connected it had also been observed that a change from a stimulating animal food, beef and mutton, had been followed by good results, and that by so doing the habit was more easily managed. The habit has been found to be excessive among the children, and when one bad child is admitted, it requires the closest watching to prevent its spread among the other children. The habit of wetting the bed and clothes had been broken up in the institution by watching the children, and breaking up the habit of masturbation. These were children from 2 to 7 year sof age."

On the Local use of Tannin.—Mr. Thomas writes to the *British Medical Journal*, on concentrated solution of tannin as a styptic. I have used it for some years, as a topical application, in various diseases, though rather as an astringent than a styptic. To prepare it of full strength, an ounce of perfectly fresh tannin must be mixed with six drachms of water, in which it readily dissolves. The solution is a thick fluid, the color and consistence of treacle, which keeps much better than tannin itself.

Most of the tannic acid found in shops contains a large proportion of gallic acid, and will not yield a very strong solution. But, if an ounce of old tannic acid be mixed with two ounces of water, a tolerably strong solution, which answers for many purposes, may be decanted off after subsidence.

The strong solution of tannin is a most powerful astringent, almost free from irritating properties. It is one of the best dressings for wounds, far superior to collodion, and even less irritating than the styptic colloid, which it somewhat resembles. If applied by a brush and allowed to dry, it soon forms a pellicle which excludes the air, and gives ease to pain. It may be applied to almost any form of ulcer, and to wonnds after amputations or other operations, especially when not very deep. It answers well, for instance, after the operation for hare-lip, painted over the pins and threads, in the same way as collodion is sometimes used.

In a female, aged twenty-six, the hair was caught between rollers and the whole scalp removed to within one inch of the left eyebrow, and two inches from the right, round on a level with the tips of the ears to about the external occipital protuberance, the periosteum being extensively removed at the vertex. There was much suppuration, followed by erysipelas. After three months, exfoliation of bone occurred, and skin grafting was performed first with eleven grafts, and six weeks subsequently with twenty-one. After varied treatment, antiseptic and other, little progress was made, till, nine months after the accident, strong tannin solution was applied. Discharge and fœtor diminished at once, and the healing process went on more quickly than before. Tenderness diminished, and the general health improved rapidly for the first time since the accident. The wound, eighteen months after the accident, was about half its original size, and the discharge trifling. The patient does household work, wears only a thin cap, and is little worse for the accident, generally or locally.

Strong tannin solution applied to the ulcerated skin of ingrowing toe-nail at once removes pain. After one application, the offending corner of the nail may be readily raised, a little lint inserted underneath, and the nail allowed to grow up. Among many cases, I have in this way cured one in which evulsion, twice performed, had proved only a temporary remedy, the disease being reproduced each time the nail grew up. For cracked nipples, this solution, diluted with an equal quantity of water is the best application, and corresponds to the tannin solution commonly used for this purpose.

Enlarged tonsils may be reduced by daily brushing with this solution. This treatment, though vastly inferior to extirpation, or even to the application of potassa cum calce, is painless, and therefore, in some cases, useful.

Bleeding warts may be readily removed by this application, as also by the percloride of iron. I have found the former to readily reduce the granulations from an unhealed umbilicus in an infant.

On Examination of the Heart.—Dr. G. W. Balfour, in the Edinburg *Medical Journal*, remarks:

From the formation and position of the heart, it is obvious that, though we can and may percuss out the whole of the cardiac dullness, this is quite unnecessary; it is only of importance to ascertain its greatest extent of dullness vertically and transversely. Increase of the vertical dullness rarely indicates any alteration in the size of the heart itself, but is usually either due to hepatic enlargement, readily ascertained by an extension of these exploratory methods to the liver itself, or to pericardiac effusion—the former dullness, as a rule, extending below the sixth rib, the latter above the third; while a simple change of position of the heart, which may arise from various causes, is indicated by a transference of the normal dullness upward or downward without any change in its extent. The apex-beat, except in several abnormal conditions, is, from the formation of the heart, the part which extends furthest to the left, and being, as a rule, preceptible to the touch, only requires to be percussed out in those exceptional circumstances where the true apex beats beneath a rib, and not in an interspace. The right auricle is, of course, that part of the heart which extends furthest to the right and being extremely dilatable, and readily influenced by any obstacles to the onward flow of the blood, transverse dullness about the level of the fourth rib comes to be an important indication of some obstacle to that onward flow, and therefore, of enlargement of the heart chiefly in auricular region. These, therefore, are the chief points in regard to which we look for important information from the percussion of the cardiac dullness. Increase of dullness above the third rib indicates, as a rule, pericardiac effusion. Increase of the transverse dullness at the level of the fourth rib indicates obstruction to the circulation. If the apex-beat be displaced to the left and downward, the obstruction is probably aortic, and has primarily influenced the left ventricle; if the apex-beat be not displaced downward, the obstruction is either mitral or pulmonary in its origin.

On the Researches of Dr. Curry, and the Recent Views with Regard to the Remedial Use of Water.—The researches of Curry were made at the close of the last and the beginning of the present century.

It appeared, from the reading of the paper, that Curry resorted to the use of water extensively in the treatment of a great variety of diseases. Not only were his observations made with regard to the effect of cold, but also of warm water. The results of his experience were extensively quoted, and the conclusion was easily drawn, that the water-treatment of to-day is essentially a revival of a practice adopted three-fourths of a century iago. It was an interesting fact, also, that Curry appreciated and clearly set forth the importance of the thermometer, and, indeed, the present axillary and self-registering thermometers were anticipated by him. The quotations from Curry, although so old, sounded like the fresh observations of a master-mind upon this subject, so fully were they in keeping with the most recent views concerning the value of this remedial agent.

THE COLD PACK.

Prof. Flint's method of using the cold pack is as follows:

Wrap the body in a sheet wet in cold water, and then sprinkle with a watering-pot, and continue the pack from ten minutes to half an hour, according to the temperature and condition of the pulse. Used in this manner he is of the opinion that we obtain all the benefits of the *bath*.

Special reference was made to the internal use of water in the treatment of disease. The quantity to be taken in the treatment of fever should be regulated by the feelings of the patient. Air, food, and drink; these form the tripod whence emanate the laws which govern the hygienic management of our cases of fever. The internal use of water was suggested in the treatment of renal affections. A case was related in which the urine was scanty, albuminous, and of very low specific gravity, accompanied by other symptoms of grave character, but was brought to a successful termination by water-treatment, originated and executed by Dr. Perry, formerly house-physician at Bellevue Hospital. The chief feature of the treatment was the administration of four ounces of water, or milk and water, every half hour, regularly and steadily persisted in.

Dr. Leale presented the records of several cases in which he had resorted to the use of water with good results.—[From a paper read by Dr. Austin Flint, at a meeting of the New York Academy of Medicine.]—*Med. Record.*

WARBURG'S TINCTURE.—No one familiar with East India medical literature can have failed to have had his attention arrested by the extraordinary accounts of the action of a secret remedy—Warburg's tincture—as a sudorific and antipyretic. Dripping sheets, mattresses soaked through, pools of sweat upon the floor, violent tropical remittents broken up as by magic, soldiers and civilians, generals and privates, saved from imminent death by this nostrum;—such has been the testimony of numerous physicians of unquestioned skill and probity. We had always supposed the inventor and proprietor of this secret remedy to be an East Indian; but it appears that Dr. Warburg is a German, or at least is a practitioner formerly of Vienna, but now of London. By the advice of friends, he has revealed his composition to Professor Maclean, who, in the London *Lancet* of November 13, imparts his information to the profession. The famous Netley, professor at the same time, speaking from large personal experience, attests the great value of the remedy, and especially its superiority to quinia. After affirming that many great engineering works in the deadly jungles of Southern India were brought to completion mainly by the aid of Warburg's tincture, he gives the following as the mode of administering and preparing it:

"'The tincture is administered in the following manner: One half-ounce (half of a bottle) is given alone, without dilution, after the bowels have been evacuated by any convenient purgative, all drink being withheld; in three hours the other half of the bottle is administered in the same way. Soon afterwards, particularly in hot climates, profuse, but seldom exhausting, perspiration is produced; this has a strong aromatic odor, which I have often detected about the patient and his room the following day. With this there is a rapid decline of temperature, immediate abatement of frontal headache—in a word, complete defervescence, and it seldom happens that a second bottle is required. If so, the dose must be repeated as above. In very adynamic cases, if the sweating prove exhausting, nourishment in the shape of beef tea, with the addition of Liebig's extract and some wine or brandy of good quality, may be required.

"In the terrible remittent fevers described by Livingstone and his followers, the form of purgative described by the great traveller, and known to his people as 'Livingstone's Rousers,' may be premised. By no authors is the necessity for careful nutrition and due stimulation so much insisted on as by the companions of this immortal explorer, notably by the Rev. Mr. Waller, who, I believe, is a physician as well as a missionary.

DR. WARBURG'S TINCTURE.

"℞ Aloes (Socota.) libram,
Rad. rhei (East India),
Sem. angelicæ,
*Confect. damocratis, ana uncias quatuor;
Rad. Helenis (s. enulæ),
Croci sativi,
Sem. fœniculi,
†Cret. præparat., ana unc. duas;
Rad. gentinæ,
Rad. zedoariæ,
Pip. cubeb.,
Myrrh. elect.,
Camphoræ,
‡Boleti laricis, ana unciam.

"The above ingredients to be digested with five hundred ounces of proof spirit in a water-bath for twelve hours; then expressed and ten ounces of disulphate of quinine added; the mixture to be replaced in the water-bath till all quinine is dissolved. The liquor, when cool, is to be filtered, and is then fit for use.—*Medical Times.*

SALICYLIC ACID (*Bulletin General de Therapeutique*, September 30, 1875).—Professor Crede has employed at his obstetrical clinic at Leipsic, a solution composed of one part of salicylic acid to twenty to sixty parts of distilled water, and a powder of one part of the acid and five of starch. He has used these as vaginal injections after delivery, in ulceration of the cervix following labor, and also as general disinfectants. The results have been so satisfactory that he particularly recommends this agent to all obstetricians.

Dr. Frontheim has tested the efficiency of salicylic acid in thirty-one cases of diptheria, none of which died, and in none of

---

* This confection, which consists of an immense variety of aromatic substances, was once officinal, and is to be found in the Ph. Lond., 1746.

† Dr. Warburg informs me that this ingredient was added to correct the otherwise extremely acrid taste of the tincture. Many other substances were tried, but none answered so well as prepared chalk.

‡ This is the Poliporus laricis (P. officinalis, Boletus purgans, or Larch agaric), formerly," Pereira, "used as a drastic purgative, and still kept by the herbalist."

which were there any renal complications. In only one case was there paralysis of the soft palate. Those patients who were most seriously attacked were cured in about eight days; the others in two, three and four days. In the more serious cases he touched the diphtheritic patches every three or four hours with a sponge fastened on the end of a whalebone and dipped in a solution of salicylic acid. After each of these applications he also administered internally a teaspoonful of the same solution, which consisted of—

Salicylic acid, grs. xxx;
Water, f drachms v;
Alcohol, q. s.

He found that the salicylic acid appeared very rapidly in the urine, and gave a bluish violet reaction with the chloride of iron.

Tincture of Capsicum in the Treatment of "Tippling."—A correspondent of *Land and Water* throws out some suggestions to alleviate, if not cure, "tippling in private life." He says: Of course, as a rule, moral means, such as persuading or frightening the patient, are futile. Dr. Ringer, in an able article in the *British Medical Journal*, in 1874, advocated the use of capsicum, "given in doses of the tincture (ten drops), before meals, or whenever depression or craving for alcohol arises." It also induces sleep in early stages of delirium tremens. It obviates the morning vomiting, removes the sinking at the pit of the stomach, the intense craving for stimulants, and promotes appetite and digestion. This treatment I have tried with great success in several cases, and one in particular—that of a young man, whom no one by any means in his power could possibly keep feom tippling. Shut up the spirits, he had a key made on the quiet, while his wife was away for a day—of course he sent her. Take away his money, he would tipple on credit. He came under my care for bronchitis. I soon heard of the propensity, and tried Dr. Ringer's treatment. I began by giving him five drops of the tincture in a little syrup of orange-peel, and some orange bitters, and increased the dose of capsicum to twelve drops. He rapidly improved, and at the end of a month he was quite another man. He was no longer to be seen in a half-muddled state, hanging about the low cabarets and taverns by himself, but every day walking out with his wife, and taking an interest in all that was going on. He left here for England about three months afterwards, and I have since heard that he still takes to his bottle (the capsicum bottle) whenever he feels inclined to indulge in the other sort of "tincture." Another case was that of lady, over forty years of age, but not so successful as the one above cited. Of course, it is a great thing to wrap up the capsicum in a convevient vehicle, and the above, suggested to me by M. Dutertre, the well-known pharmacien of our town, is, I think, as good a form as any.—*British Medical Jourual.*

Bromide of Lithium (*The Doctor*, October 1, 1875).—The *Revue de Therapeutique* gives the following as the result of Dr. Rouband's test of this drug:

1. Bromide of lithium is a drug which has a twofold action.
2. It possesses in a high degree the lithontripic qualities which are universally recognized in the salts of lithia.
3. It affects reflex sensibility in a more energetic manner than the other bromides, without the unpleasant effects on the heart which the bromide of potassium has.
4. Consequently, it takes its place in the first rank of antilithic and sedative drugs, and its action is especially valuable in cases of the uric acid diathesis which are accompanied by painful phenomena.—*Medical Times.*

A Powder for the Treatment of Coryza (*Le Progress Med.*, October 2, 1875).—Van der Corput recommends that the following power be employed once or twice daily in cases of obstinate coryza:

℞ Subnitrate of bismuth, ℥ ii;

Powdered benzoin, ℥ i;

Muriate of morphia, gr. iss.

Mix, and make two powders.

Sulpho-carbolate of Sodium as a Prophylactic in Scarlatina.—Dr. W. Scott writes to the editor of the *Medical Press and Circular*:—Without, on the present occasion, entering into the interesting question of whether the minute particles of living matter which constitute disease germs consist of animal or vegetable bioplasm, or the various other difficult points regarding the nature of the poisons of infectious disease, I am desirous to direct the special attention of practical and thoughtful men to the use of the sulpho-carbolate of sodium, both as a curative and prophylactic agent in the treatment and cure of scarlatina. So far as I am aware, there has not been that general attention given to the subject which its vast importance demands. Mr. Crookes, Dr. A. E. Sansom, Dr. Brackenridge, perhaps others, deserve well for what has been already effected, and, so far as my experience goes, I can fully confirm their testimony; but unless a considerable number of the medical profession carefully attend to the matter, noting cases and briefly publishing results, little partaking of certainty can be reliably settled.

In scarlet fever, diptheria, and measles, the sodium sulpho-carbolate may be given in doses varying, according to age, from five to thirty grains four times daily, and in a mixture with simple syrup, or other such ingredient, is in no way disagreeable. An extended trial is the all-important point at present, and for this time I need not only say that I have seen the use of the above named preparation followed by results far exceeding my most sanguine expectations, both in a preventive and curative point of view.—*Medical and Surgical Reporter.*

NITRITE OF AMYL IN GASTRALGIA.—Dr. Forrest says: I first tried the nitrite of amyl by inhalation, and, obtaining a slightly relaxing effect only, gave the drug by the mouth, in a dose of *m* ijss. The first method, although exhibiting most of the characteristic effects of the medicine, failed to stop the pain, which end, however, was obtained by the second, in precisely eight minutes. The patient walked off, declaring that he felt peculiarly strong. The spasm, thus cut short, remained absent for about twenty-four hours, but then returned in full vigor. The experiment was again resorted to in like manner as at first, the dose by the mouth being increased to *m* iijss. The pain ceased as before, but has never since returned to an extent sufficient to cause anything but a slight annoyance.—*New York Medical Journal.*

Obstinate Intermittent Fever Cured by Steam Bath. Dr. Finkelstein, of Jassi, in Roumania, states that intermittent fever is exceedingly prevalent in that country, and that while most cases yield to quinine in large doses, and to arsenic, obstinate cases sometimes occur which defy all ordinary treatment. He instances his own case as one of these. First attacked, in 1865, with a quartan, he was benefitted by quinine, but had relapses whenever he stopped taking it, until he went to the mountains. He was then free from it for six years, until 1871, when he settled in Jassi. From this time until 1874 he was constantly subject to attacks—the paroxysms lasting from seven to eight hours. He took quinine and Fowler's solution, which had good temporary effects; also piperin, carbolic acid, and tincture of eucalyptus globulus, all without effect. On September 4, 1874, he had the premonitory symptoms of a chill, and it occurred to him that as the process always ended in perspiration, if he could induce free action of the skin an earlier defervesence might set in. He determined to take a steam bath at once, and before he could get his clothes off his teeth were chattering. His pulse was then 96. On entering the hot chamber its temperature was 102.2° F. He directed the heat to be increased, and when it reached 105° he began to have an agreeble feeling of warmth, and broke into a perspiration. Pulse then 100. After fifteen minutes, beginning to feel some oppression in the chest, he left the hot chamber and took a cold douche. Finding, however, that the chill had not left him entirely, he returned to the sweating room, the temperature of which had meanwhile risen to 107.6°. He remained there for twenty-three minutes longer, the temperature of the room reaching 111.2°. At this time he began to feel dizzy, and at once left the hot room, had himself well rubbed and douched, and at the end of the process, which lasted nearly an hour, he had no chilly feelings, and his pulse was 80, sinking to 70 after he was dressed. Six months had elapsed when he wrote, and he had no return of the disease, and had taken no medicine. Dr.

F. reports equally good success in the treatment of a similar case, in a patient of his, whom quinine and arsenic would not cure.—*Berl. klin. Woch.*, Sept. 6, 1875.

Carbolic Acid in Diabetis.—At the session of the General Surgical Society at Cologne, held on February 15, 1875, Boese reported a series of observations made on a case of diabetes mellitus, which had been treated with carbolic acid. A quantitative estimate was made daily of both the sugar and urea. The patient was kept on an abundant ordinary mixed diet for sixteen days; then for fourteen days on a strict diabetic diet; and then for a further period of thirty-one days, while on the same diet, carbolic acid was given in the way recommended by Ebstein and Miller. For a further period of fourteen days the carbolic acid was suspended, while the same diet was continued. The patient then took the acid again until discharged. It appeared from the analyses that on beginning the diabetic diet the quantity of urine and of sugar diminished, and remained so while the amount of urea continued at about the same figure. On giving the carbolic acid, the amount of urine and of urea remained unchanged, but the sugar diminshed by one per cent. to increase by that amount when the acid was discontinued, and diminish again when it was resumed.—*Berlin. klin. Woch.*, September 13, 1875.

A paper on rectified spirit, (*spiritus frumenti rectificatus,*) by A. W. Miller, M. D,, Ph. D., is published in the *American Journal of Pharmacy* for November, 1875. The writer thinks that pure rectified spirit possesses merits and advantages which heretofore have not been properly appreciated by physicians. "French spirit," "sweet liquor," and "rectified spirits," are terms used by the liquor trade to designate pure rectified whisky freed from fusel-oils, coloring matter, and other impurities. It is obtained by percolating the ordinary raw corn whisky through fresh charcoal. It is the basis used by compounders of fancy liquors for their cordials, etc. All the various fusel-oils in a concentrated form have peculiarly penetrating and oppressive odors, but it is on these in various proportions and admixture that the distinctive flavor of different liquors depends. It is by no means certain that the medicinal virtue of spirit is enhanced in the smallest degree by the costly flavors which characterize the choicest cognac, Jamaica rum or Bourbon. In the plain, rectified spirit, however, we possess a liquor of almost absolute purity, which deserves to be regarded as the type of a simple arterial stimulant. It can be obtained everywhere with facility, of standard and uniform strength, and at a fraction of the price of the fancy flavored liquors. The conclusions are the following: Rectified spirit is almost always strictly pure, while the more expensive liquors invariably contain fusel oils, and frequently other impurities. The market price of rectified spirit is at present from $1.25 to $1.50 per gallon, that of the fancy flavored liquors from $2.50 to $12.00. While the taste and odor of rectified spirit is not so tempting as that of the choice cabinet liquors, it is entirely free from the disgusting taste and smell of ordintry diluted alcohol. It has not yet been established that the more expensive liquors are in any way superior to rectified spirit, or that their physicological action presents tangible points of difference.—*Boston Medical Journal.*

THE FORM OF ALCOHOL TO BE USED MEDICINALLY.—The above question is discussed in the American *Journal of Pharmacy*, by M. A. W. Miller. He says:—

Raw corn whisky or high wine, such as is used for the manufacture of alcohol, is undoubtedly strictly pure, as there is no incentive whatever to its adulteration. Nevertheless, many vile epithets, such as "Jersey lightning," "rot-gut," etc., are heaped upon this, simply because it is lacking in smoothness, oiliness, and body: so that it meets with little favor among those who are sufficiently familiar with it to recognize at once its want of age.

In the asthenic forms of many diseases, it is of prime and often of even vital importance to administer alcohol. Nothing as yet known so well substitutes the functions of food, and thus bridges over the chasm of greatest prostration, during which the system would otherwise inevitably succumb.

While we cannot and dare not dispense altogether with a drug of such inestimable value, what is there to be gained by running the unnecessary risk of inculcating a taste for the truly fragrant bouquets of choice French brandy, or the almost equally precious old Kentucky Bourbon? We can well afford to dispense with this meretricious and alluring *haut gout* of liquors, which, even in their purest state, are but too apt to win boon companions, ready and willing to follow their enticing solicitations.

The economic aspect is another strong point in favor of the introduction of plain rectified spirit into use as an officinal medicine. Why should the poor day laborer, suffering, perhaps, from typhoid fever, or, it may be, pulmonary phthisis, be compelled to devote his entire compensation for two or three days of hard toil to the purchase of a bottle of pure imported brandy, when the value of an equal amount of pure spirit, from which he will derive quite as much benefit, can be earned in as many hours?

We may sum up as follows: Rectified spirit is almost always strictly pure, while the more expensive liquors invariably contain fusil oils, and very frequently other impurities. The current market price of rectified spirit at present is from $1.25 to $1.50 per gallon, that of fancy flavored liquors ranging from $2.50 to $12. While the taste and odor of rectified spirit is not so tempting as that of the choice cabinet liquors, it is entirely free from the disgusting smell and flavor of the ordinary diluted alcohol.

Chloroform and Morphine.—As long ago as 1864 Bernard discovered accidently the valuable form of anæsthesia that could be produced by *combining the effects of chloroform with those of morphine.* The very same week the discovery in the laboratory was confirmed by a clinical observation of Nussbaum; and to-day this combination is frequently utilized in practice. In his new lectures, Bernard discusses the theory of the combined action of these two powerful agents. It is known that after a small dose of morphine (½ centigrammes) by subcutaneous injection, the inhalation of a very small amount of chloroform suffices to determine anæsthesia. Conversely, an injection of morphine at the moment that the effects of chloroform are about to pass away will renew anæsthesia, much more profound and prolonged than would be caused by a single fresh inhalation of chloroform. In the first case, the morphine has begun to destroy the physiological properties of the nerve element, thus rendering them more excitable, as are all elements when beginning to die. In this state they are susceptible to a far less degree of vitiation of the blood by chloroform than when in a normal condition, and hence succumb much more rapidly to its influence. In the second case, as the anæsthesia is beginning to disappear there yet remains some chloroform in the blood, and this amount, insufficient to suspend the properties of sensitive elements in a normal condition, becomes sufficient when they are submitted to the action of morphine. Hence, two agents acting upon the same elements, but in a way which, if not antagonistic, is at least entirely different, may so combine their effects as to produce a result similar to what would have been obtained by a more powerful and dangerous dose of one of them alone.—*Virginia Medical Monthly.*

IN STRYCHNIA POISONING, chloral has been found more successful than in spontaneous tetanus. In a case quoted in the *Revue Therapeutique*, June 15, paroxysms of opisthotonos were occuring every 30 or 40 seconds. After administration of 1 grm. 80 centigrammes of chloral, the paroxysms ceased, but returned in 30 minutes. After a second dose, a new pause of 50 minutes; after a third, only one slight access occurred in the course of 3 hours; and after a fourth, the patient fell asleep and awoke cured.

In the report of the Edingburgh committee, appointed to investigate the antagonism of various poisons with one another, the mutual action of strychnia and chloral was carefully tested. (*Brit. Med. Journal*, vol. 2, p. 436, 1874.) The average fatal dose of chloral was shown to be for a rabbit, 21 grains to every 3 lbs. of its weight; for strychnia, $\frac{1}{96}$th of a grain. This ascertained, it was found that the simultaneous injection of $\frac{1}{90}$th grain of strychnine, and 15 grains of chloral, was followed by recovery; or, in other words, a non-fatal dose of chloral, if given at the same time as a fatal dose of strychnine, was able to avert death. If the administration of chloral followed that of strychnine in from 2 to 8 minutes, it was equally successful as an antidote; but was useless if given later than 10 minutes from the time of poisoning. A fatal dose of chloral, however, was little modified by strychnine. The committee consider these facts of immense importance for the treatment of strychnine poisoning, tetanus, and other spasmodic diseases.—*Va. Med. Monthly.*

CHLORAL AS A LOCAL APPLICATION FOR ULCERS.—A writer in the *Lancet* says:—On visiting the wards of Guy's Hospital, we saw several patients on whom a solution of hydrate of chloral had been used as a local application to ulcers, and the results appeared to be sufficiently satisfactory to be worthy of record. Mr. Lucas commenced the use of chloral among his out-patients in August last, for cases of sloughing wounds and fetid ulcers, and, being pleased with the result, he has since given it a somewhat extensive trial in the wards. The effect of the local application of chloral appears to be that of a powerful stimulant and disinfectant; it has no soothing or sedative effect upon the part to which it is applied, but, on the contrary, gives rise to considerable pain, which lasts some time; nor does it, even when used over a very extensive surface, ever become absorbed in sufficient quantity to act as a hypnotic. Whether it is taken up into the circulation or not, matters little, since the quantity used as a local application is so small, compared with the dose administered as an internal remedy, that were the whole of the drug applied to find its way into the blood, the quantity absorbed would still be very much less than that of an ordinary sleeping draught. Its local application is, therefore, eminently safe and free from the dangers which sometimes follow the use of opium lotion or carbolic lotions long continued. Mr. Lucas has used solutions of various strengths, that which he has found most useful being a solution of four grains of hydrate of chloral in an ounce of water. The application of a lotion of this strength is, as we have just stated, often attended with considerable smarting, which may last a quarter of an hour, but the smarting becomes less at each subsequent application. In cases where the patients have complained much of the smarting, the lotion has been diluted to the proportion of three or two grains to the ounce. The treatment of foul sloughing ulcers by means of chloral lotion has been attended with great success, the surface of the sore quickly cleansing and as- suming a healthy appearance, whilst the subsequent healing has advanced with a rapidity in some cases quite astonishing.

Under the use of chloral lotion the ulcers quickly became sweet and clean, and the cuticle spread over them with very great rapidity, even while the surfaces of the sores were still considerably below the level of the surrounding skin. The smaller ulcer was completely healed within the fortnight, and at the time we saw the patient all that remained of the large one was a small fusiform patch of healthy granulations, about half an inch in breadth and two inches in length, upon which a blue line of cuticle was rapidly encroaching.—*M. S. Reporter.*

SULPHURIC ACID IN DISEASED JOINTS.—At Guy's Hospital, London, says the London *Medical Times and Gazette*, the treatment of diseased joints by sulphuric acid is likely to be fully tried. Mr. Cooper Forster has resorted to it in one or two cases, and some weeks ago we had the opportunity of seeing the method applied by Mr. Durham.

Three patients were thus treated on this occasion, all of whom were suffering from advanced pulpy degeneration of the synovial membrane of a joint, and in one case a condyle of the femur was also necrosed, and a small piece of bone was removed from the joint with forceps. Four joints in the three patients were operated upon; in one patient the ankle-joint, in one the knee, and in one the knee and wrist-joints were the seats of disease. The mode of operating was simply to make one or more incisions into the joint, let out any flaky or puriform fluid which might be present, and then to stuff strips of lint soaked in a mixture of one part of the sulphuric acid of the shops with two of water, into it. The parts were then covered with carded oakum and a bandage, and the limb was fixed on a splint. No great amount of pain can be produced by the application, for one of the patients seemed quite comfortable and without any sign whatever of suffering, when we saw her again, about half an hour after recovering consciousness from chloroform.

Through the courtesy of Mr. Howse we saw, at the same visit, several cases of excision of the knee-joint which had been operated upon by him. Mr. Howe operates in the usual way, but his method of immediate after-treatment is somewhat different in some of its details to that which is generally followed elsewhere. After the operation is finished, and before the patient leaves the operating-table, the limb is set in a modified McIntyre splint, such as has been before described in these columns as in use at other institutions; then the spaces between the sides of the splint and the limb, are filled with cotton-wool soaked in melted wax; next a roller soaked in the same material is applied around the limb, and splint above and below the wounded parts. The wound itself is treated strictly on Lister's plan, except that sixteen thicknesses of gauze, instead of eight, are used, but no oil-silk or mackintosh. Another point is that one-third of the mattress is removed from the footboard end of the bed, and the cradle for swinging rests immediately upon the corded webbing of the bedstead. This much facilitates the dressing of the wound, and does away with the inconvenience of draw-sheets, while the other limb rests quietly upon a pillow raised to the same level as the mattress itself.

By adhering to this method, Mr. Howse has had excellent results with respect to limb as well as to life. The mortality, indeed, has been very small, for out of twenty-seven cases he has lost but two—one patient died of pyæmia, whilst ulceration sloughing were going on about the leg not operated upon, and after firm union had taken place between the bones at the seat of the excised joint; whilst the other died of tubercular meningetis, and had tubercle deposited in several of the abdominal viscera.

THE COMMUNICATION OF TUBERCLE IN FOOD.—The Edinburgh *Medical Journal* states that Professor Gerlach, of Berlin, details an elaborate experimental research on the question, whether tubercular matter, or the flesh of tubercular animals, can communicate or excite tubercular disease if taken as food? The method employed by Gerlach was to introduce into the stomach of the animal one or two doses of tubercular matter.

The effects, if any, were observed; and if the animal did not die from these, it was killed some weeks or months after the administration of the substance, and a post-mortem examination was made. Great is the importance of the inquiry, not only from a hygienic point of view, but also as relating to the etiology of tubercle, and its transmissibility in the human race. The conclusions arrived at by Professor Gerlach may be summarized as follows:—

1. There is a specific virulent material in tubercle, and many of the symptoms of tubercular disease are due to the absorption of this virus.

2. This virus exists in tubercle in all its stages, but apparently in greater intensity in cheesy masses. It is found in recently formed tubercle, and miliary tubercle.

3. The infection begins first in the mucous membrane of the mouth, and if the tubercular matter be in contact a sufficient length of time with the mucous membrane of the alimentary canal, it may communicate the disease to the whole lymphatic system.

4. While tubercular disease has special characters in different animals, all tubercular matter, when introduced into the alimentary canal from one species to another, is more or less virulent.

5. The tubercular matter of birds, especially that of the common hen, is very virulent, and is identical in its action with that of mammalia.

6. The fibrous tubercle of horses, without a trace of cheesy formation, is just as infectious as the miliary tubercle of cattle.

7. The flesh of tubercular animals is also infectious, though in a much less degree than tubercle itself.

8. Tubercular material cooked for a quarter to half an hour is still infectious, though in a much less degree than that not cooked.

9. The effects of poisoning by tubercular matter taken into the alimentary canal are irritation of the mucous membrane, both of the alimentary and respiratory tracts, enlargement and tendernsss of the lymphatic glands, enlargement of the bronchial glands, and the formation of tubercle in the lungs and other organs.

THE USE OF COLD IN SCARLATINA.—In the *Lancet*, September 4th, Dr. J. T. Eddison says, quoting several cases showing the value of cold in this disease:

All sorts of objections have been, and still are, urged against the use of cold in scarlet fever. Danger is said to arise from "driving in the rash," from internal congestion, from the rapid loss of body temperature and consequent depression and it is also said that the risk of renal mischief is thereby increased. If it were proved that the occurrence of nephritis is more frequent after treatment by cold, it would be a very valid objection to the practice of it, and the real truth can only be learned by an examination of a large number of cases. From my own experience, I am inclined to disbelieve that any harm results in this way. The fear of the occurrence of nephritis from this cause originates, no doubt, in the generally accepted opinion that the affection of the kidneys so commonly occurring after scarlet fever is due to "draughts," or "catching cold," or to leaving bed too soon. I think this opinion is not founded upon sufficiently good grounds, and that every one in the habit of seeing cases of scarlet fever must often have seen nephritis beginning before, as well as after the patient has left his bed, and as often in cases kept in warm stuffy rooms, as in those in which fresh cool air has been freely admitted to the sick chamber. "Catching cold" is made to do duty as a cause for so many conditions for which we can find no better explanation, that it is adopted at once and without hesitation, in order to account for any otherwise inexplicable phenomena. There appears, at any rate, to be no good ground for assuming that cold bathing increases the chance of an attack of nephritis, and in the two cases here reported the urine did not become albuminous after repeated bathings. Objections on the ground of the trouble and increased expense in nursing are scarcely worth consideration, if it be true, as I believe it is, that this mode of treatment is better than any other. The difficulty is perhaps less in treating scarlet fever than in dealing with other cases, because the patients are usually young and easily lifted in and out of bath, but when, from the weight of the patient, or the weakness of the attendants, it is impossible to use the bath, the patient may easily be packed in wet sheets, with or without pieces of ice placed here and there, or india-rubber bags, or large bottles filled with ice, may be placed around the patient. The bath gives better results than any other plan, when it can be thoroughly carried out, and the most satisfactory way is to begin with the bath at ninety-eight or one hundred degrees, and cool down gradually to about seventy. It is of course, better that the temperature of the patient and of the water should be frequently taken, but the hand is generally a good enough guide as to the water, and the appearance of the patient always indicates the improvement in his condition. This is well illustrated in the case of S. F—— (aged eight), near the end of the fifth day. The little patient was then drowsy and delirious, the temperature being one hundred and four degrees, and three-quarters of an hour in a bath, beginning at ninety degrees and cooled down to sixty-eight degrees, resulted in the cessation of delirium and drowsiness, and a reduction of the temperature (in axilla) to 95 degrees. In the case of M. A. S—— (aged four), a similar bath for one hour reduced the temperature from 105.3 degrees to 95.6 degrees. There was no dangerous depression, or bad symptom whatever, from this low temperature. In scarlet fever, as in other allied disease, the cardiac impulse, and the character of the heart sounds are safer guides as to the condition of the heart and circulation, than is the pulse at the wrist, the latter being often very deceptive.

Sprain.—Professor Levis says: When a patient is brought to you for diagnosis, hold the foot firmly in the left hand, and with the right hand make firm pressure upon the upper part of the fibula; if the patient flinches and cries out with pain, and if, on further examination, you find some lateral play of the joint, you have the certainty of tracture of the lower end of the fibula, even without crepitus, which is not always easily made out in this situation. If, on the contrary, pressure on the fibula does not add to the pain of the patient, you will direct your attention to the joint, and ask yourselves if you have to deal with injury of the ligaments, or of the tendons, and if the swelling be due to effusion into the joint, or into the sheaths of the tendons. I am convinced that stretching of the tendons of the flexors and extensors is a frequent condition, too often ignored. They are especially liable to sprain where they curve, the extensors around the external, the flexor around the internal malleolus. Diagnosis is made by putting the injured muscles upon the strain. In the case before us, you see the patient flinches from extension; we are, therefore, sure we have sprain of the extensor tendons. Have we also effusion into the joint? There are two points where the joints is comparatively superficial, at the inner and outer sides of the extensors. Looking at these points we find the hollows naturally existing effaced, and pressure, even slight, gives exquisite pain. Our diagnosis is made We have here sprain of the joint, with effusion into the joint, with sprain of the extensor tendons. In regard to treatment, a recent sprain is best treated by hot water. Put the foot in a bucket of hot water, kept as hot as can be borne for half an hour. Just how this acts we cannot say, but there seems to be more than a revulsion of circulation. We may then dress with an ordinary bandage, or better, some fixed dressing which shall keep the parts at rest.—*Medical and Surgical Reporter.*

STOMATITIS MASERNA.—Dr. J. B. Hoag writes to the *Journal of Materia Medica:*

I have recently come in possession of a prescription, which, from the testimony of those who have repeatedly used it, and in whom I have most explicit confidence, as well as from my own experience and observation, I believe to be, when thoroughly and judiciously used, as nearly a specific in this complaint as it is possible for any medicine to be in any disease.

The prescription is as follows:

| | | |
|---|---|---|
| ℞ | Biniodide of mercury, | grs. v |
| | Iodide of potassium, | grs. x |
| | Water, | oz. j. M. |

Dose, gtts. 3 to 6, three times a day, after meals. For topical use, add 6 drops to a tablespoonful of water, and wash the mouth thoroughly three or four times a day.

It is of great importance that the water should contain no alkaline properties, hence the necessity of using distilled rain, river, or other "soft" water.

It should be given after meals, as, when given on an empty stomach, it is liable to nauseate.

Duration of Bloodless Operations.—At a recent meeting of the fourth Congress of the German Surgical Society, Prof. Langenbeck stated his opinion that it was of extreme importance to determine more accurately than has yet been done how long a limb can be deprived of blood, during a bloodless operation, without danger either to it or to the patient. He thought that the constriction could be kept up for a very long time without fear of gangrene, if the patient could only overcome the disagreeable sensation which the bandage caused. We, however, particularly need experiments on animals, to determine the exact limit of safety. In long operations on the bones—for example, resections—we can see that the bloodlessness is not complete in them as in soft parts around, and that capillary bleeding occurs from them when not a drop of blood issues from the muscles. Prof. Langenbeck continued: "I have recently kept up the contrition in excisions of the ankle joint for an hour and a half, without any ill effect. After this operation I always find a plaster-of-Paris bandage the most comfortable and useful dressing; but still lately there was one great disadvantage attending its use—namely: that it became soaked with blood as soon as it was put on. In the last two cases in which I have operated, I have kept up the compression until the bandage was perfectly hard, and then cut large openings in it until the whole wound could be seen, and only then removed the constriction. The abundant hemorrhage which now occurs from the vessels of the periosteum and the bone soon stops, if the femoral artery is compressed for a short time; with a little care the blood can thus be kept from soaking into the bandage."—*Medical Times and Gazette*, July 31, 1875.—*Buffalo Medical and Surgical Journal.*

Hydrastin in Gonorrhœa.—Dr. J. N. Bredin, in the *London Lancet*, says: "As far as internal treatment is concerned, I merely give in the first stage a saline aperient to be continued three times daily for four or five days, together with the followgin injection: hydrastin, one drachm; solution of morphia, (Magendie's), two drachms; acacia mucilage to four ounces; to be used three times daily. This I have employed when inflammation ran very high, without even the slightest ill effects, and have used it in every stage of gonorrhœa with the most beneficial results, when every other treatment, both internally and locally, had failed, including red sandalwood oil. But there is one remark I wish to make regarding the use of injections, which medical men generally forget, and that is, to tell their patients to micturate previous to its use. Unless this is done, injections in gonorrhœa are useless. Hydrastin is used very much in different parts of the United States, and very successfully. My last patient was a farmer who had a gleety discharge for seven months. His medical man had quite wearied him out with injections, etc., all to no purpose. I at once tried the hydrastin, and in two weeks he was quite well."—*Chem. Gazette.*

TREATMENT FOR HYSTERICAL ATTACKS.—According to M. Charcot, who has lauded this method very highly, compression made with the hands over the abdominal region corresponding to the ovaries, will almost instantly arrest the most violent attack of hysteria.

In this regard, Dr. Caffe reports, in his journal, the following interesting facts:

More than thirty-five years have passed since I witnessed, with Professor Chomel, a most violent attack of hysteria in a young woman of high social position, thwarted in her marriage projects. The very learned clinician (who was at that time the family physician) immediately advised me to make compression with both hands in the iliac fossæ of the patient. The attack promptly subsided through the compression of the aura hysteria.—*La Tribune Medicale.*—*Lancet and Observer.*

A NEW SALVE FOR THE ITCH.—R Styrax; flor sulphur; cret. præpar. *aa* 16 grms.; * green soap; † axung *aa* 32 grms. M.

This ointment is of a greenish-yellow color, of good consist-

* 1 gramme equals 15⅜ grs.

† Soft soap of the druggist.

ence, and of an agreeable odor. The patient applies the ointment at night before going to bed, taking good care to rub it in thoroughly over those parts where the acari are usually found. These inunctions are repeated for two consecutive days; the patient can go about his business through the day; at the end of three days the patient takes a full bath. For infants at the breast, an equal part of simple cerate is added to the ointment.—Dr. Weinberg, in *Wiener-Medic. Wochenschr.*—*Lancet and Observer.*

Chinoidine Pills for Chills :—

| | | |
|---|---|---|
| ℞. | Chinoidine.......................................... | 1 ounce. |
| | Ext. Colocynth.... ................................ | 1 drachm. |
| | Oil Black Pepper.................................. | 1 drachm. |
| | Carb. Iron, Precip................................ | 1 drachm. |
| | Rhei Indicum...................... ................ | 1 drachm. |
| | Quinia Sulph....................................... | 1 drachm. |

Mix—Pill mass. Div., and make 360 pills.

Those pills should be sugar-coated, in order to have them retain their pill form during the warm months of the year.

For an adult, who is laboring under any form of intermitting fever, I usually direct the patient to commence twelve hours before the paroxysm, taking two pills every four hours, until six have been taken; and so repeat, commencing before each expected paroxysm, and so continue, from day to day, until the disease is broken up.

In order to prevent a relapse, (as happens with those cases treated with Sulph. Quinia exclusively,) the pills should be repeated, at least, once a week, the same as if the fever was expected. By so doing, the impoverished condition of the blood is so improved, with respect to its normal healthy condition, that the system is completely fortified against a return, and a cure completed.

Nitrite of Amyl in Hiccough.—Dr. Simon, of Chicago, reports in the Chicago *Medical Journal and Examiner* that a patient who had hiccoughed for twenty-six hours, and been subjected to various treatments without success, was relieved almost instantaneously by inhaling three drops of the amyl.

COLLODION IN TREATMENT OF CARIOUS TEETH.—Dr. Lardier advises, in *L' Union Medicale*, to drop some collodion into carious teeth, after the latter have been cleaned and dried. The liquid collodion exactly fills the cavity of the tooth; the ether evaporates, and narcotizes the nervous twigs. By solidifying the collodion protects the tooth from the contact of the air. Dr. Lardier states that he has thus succeeded in relieving many patients.

USE OF APOCYNUM CANNABINUM IN DROPSIES.—Dr. Walter Lambert writes to the *Canada Lancet* that during a practice of nineteen years he has often prescribed milk-weed for the relief of dropsies, and with success. He says: "I always employ the root, and have it dug fresh from the earth, and advise a strong infusion to be made, of which the patient is directed to drink freely, so as to procure two or three watery evacuations from the bowels during the day. It is true that it has sometimes failed to meet my expectations and wishes, but this might be partially accounted for by the root hot having been gathered at the proper season of the year; and in some cases its failure has doubtless been due to the oiigin of the malady. I have found that it is more likely to prove of service in hepatic and cardiac dropsies, and its virtues to be owing to its alterative action on the glandular system, as well as its hydragogue properties, whichwill not fail to put in an appearance when the drug is administered in sufficient doses.

STYPTIC COTTON IN THE TREATMENT OF OTORRHŒA.—Dr. Thomas G. Morton, of Philadelphia, writes to *The Medical Times* that he has used styptic cotton wrth excellent results in a number of cases of dischaige from the ears.

The styptic cotton is prepared as follows: Take a roll of fine jeweller's cotton and thoroughly saturate it in a mixture of Monsel's solution of the subsulphate of iron, diluted with two parts of water; let it stand in the mixture for forty-eight hours; press the liquid out, and dry in a warm room, then pick or card out in fine shreds. It is better to make it in small quantities, as there seems to be some change in the cotton when kept for any length of time; losing its texture and breaking up in a fine powder when handled, thus rendering it unfit for application. In the case cited, the first plug was removed the day following its insertion, showing a healthier condition of the metus, with marked lessening of the discharge; all the pus was absorbed by the cotton, which left the drum and canal quite clean; at the end of the first week only a slight moisture remained, and in a fortnight the cure was complete, and has so continued; the opening in the tympanum closed up at the same time.

Encouraged by success in this instance, he has since used no other treatment in a very large number of cases of otorrhœa, and always with the same excellent result.

Before using the cotton, the ear should each time be thoroughly cleansed with tepid water, with a Davidson's syringe, and the canal then wiped out with common cotton; then a portion of the "styptic cotton" should be rolled upon an instrument having a screw shaped extremity, an illustration of which accompanies the original article. The plug of cotton being at least the length and width of the canal and carried in until the pulg rests close to, but not in contact with, the drum; by reversing the screw movement the instrument is easily withdrawn, leaving the cotton behind.

The first few plugs should be changed daily, then, according to the lessening of the discharge, not so frequently.

He has not observed any trouble or pain arise from the use of the styptic cotton, while it usually has an anodyne effect.

[In preparing this cotton, a precaution not mentioned by Dr. Morton consists in carefully cleansing it with caustic soda to remove adherent oil, and subsequent washing to get rid of the alkali.]—*New Remedies.*

The Diagnosis of Secondary Heart Affections.—In an article in the *Lancet,* Dr. J. M. Fothergill says:

In forming a diagnosis of secondary affections of the heart, and determining that the heart symptoms are really consequential to some prior and casual disturbance of the circulation, there are several points deserving of special notice.

1. The attacks of dyspnœa, palpitation, or sensation of heart stoppage are not induced by effort but come on independently of any exertion. They not uncommonly come on during great quiet, and even when lying in bed. Not rarely they permit of active exertion, which does not tax the heart in this class of cases; while in primary affections of the heart effort always—or almost always, to be very safe—reveals the existing adynamy. A little active movement will render the evidence of true heart failure more pronounced, as I remember well in an old man who used to take a sharp turn around the house to illustrate how exercise made his heart palpitate; while in pure cases of secondary heart affection exertion does not induce the attacks.

2. The pulse is not that of cardiac debility; even when the pulse is very irregular there is a force about it which resists compression, quite different from the irregular compressible pulse of cardiac dilitation. There is indeed the sustained pulse of high blood pressure in the midst of the irregularity, or intermittency. Not rarely, if the arteries are atheromatous, the irregularity is found along with a full sthenic and powerful pulse—a combination, once well noted, ever afterwards recognizable, though not described by words. It is a matter of no slight moment to become familiar with the pulse of these conditions. The feeling of the pulse here is not a mere formal matter, but a measure which often gives valuable indications and directs the line of investigation to be pursued.

3 The first sound of the heart is good usually—loud, clear, and of fair tensity. It preserves its muscular sound, and is not simply valvular, and does not approach the second sound in character. When there is much hypertrophy, the first sound is loud and long. Not uncommonly there is some reduplication of the first sound from delay in the contraction of one of the ventricles. On examination of the heart, it is found acting well and not giving evidences of lack of power. The rythm may be disturbed; but there are not evidences of heart failure accompanying the disturbed rythm.

4. The second sound of the heart is accenturated. This is a most important diagnostic point. In primary disease or debility of the heart, this symptom is never found; in the forms of heart trouble which are the consequences of some prior disturbance of the circulation, causing a rise of blood-pressure in the arteries, this objective symptom is never wanting. The accentuation of the second sound will, of course, be more or less intense, according to the rise in the blood pressure. It may be found at both the pulmonary and aortic valves. It is most distinct, however, at the aortic valve; and heard most loudly at the second right costo-sternal articulation. The semi-lunar valves are closed by the recoil of the elastic arteries; if then the tension in the arteries is increased, these valves will be closed with unwonted force, and their closure produces an accentuation of the normal sound. Such accentuation is ever found in the pulmonary valves when the mitral valve is diseased; here it is the measure of the engorgement of the pulmonic circulation. When heard at the aortic orifice this accentuation may be associated with aortic aneurism, with general paralysis of the insane, or with the general rise of blood pressure in lithiasis. It is easy to separate these conditions. The Germans attach great importance to this objective symptom, as might be supposed from their general high estimate of signs whose existence and explanati n are clear and rational. If the second sound is produced by the recoil of the elastic arteries, when the blood pressure is abnormally high this sound will be unusually loud.

THE CATHETER IN ENLARGED PROSTATE.—At a meeting of the Tri-States Medical Association, held at the Delaware House, New York, December 1st, 1875, Dr. W. L. Appley, of Cochecton, New York, read a paper on the treatment of enlarged prostate; advocating the early use of the catheter as the best remedy; giving his experience, and the result of several cases treated by him in this way. Three men over sixty years of age, that had acquired the tolerance of the catheter, and introduced themselves four or five times daily, and could not void a drop of urine without it, depend upon it entirely to empty the bladder. One had used it two years, one four or five one had worn a silver catheter, constantly, for ten years, removing it once a week, long enough to wash it. The man is hale, ruddy-faced and quite active. The Doctor urged it as a duty upon the profession, to be unanimous in insisting upon the early use of thc catheter by the unwilling patient; and have him understand the importance of commencing the use of the instrument early, and have him learn to be his own surgeon, and acquire the tolerance of the instrument before he has retention.

The Association voted, unanimously, to have the paper published.—*Medical and Surgical Reporter.*

PURITUS VULVA.—Dr. Gill—(*St. Louis Medical Journal*) writes: The nitrate of alumina has given more satisfaction than any other remedy, or combination, I have prescribed in quite a number of cases.

Indeed, patients have repeatedly told me it acted like a charm, relieving them immediately, and that they could not do without it.

The form in which I have used it is from four to six grains to the ounce of soft water, using it as a vaginal injection, or external wash, I ordinarily direct the patient to take a teaspoonful (beginning with rather less at first) of the powder, put it into a pint or a pint and a half of soft water, and use as a wash, or vaginal injection once, or if necessary, twice a day. In hot weather it may be needed twice a day.

The remedy is not to be obtained of all druggists; I have found difficulty at times in procuring it. This is the only objection I have found to it.

THE COLD WATER TREATMENT.—Dr. Foltz, of Lyons, has lately published, in *Lyou Medical*, a valuable series of papers, containing the results of his employments of cold water injections in typhoid fever. He states that they exert a general and local action. The local action consists in a cooling sensation, accompanied by intestinal contractions. The general action produces slackening of the pulse, marked diminution of temperature, and quietude of the nervous system. It abates thirst, excites the appetite, and increases the secretions. The cooling, sedative, and tonic action is about the same with all injections under 38 degrees (Centigrade); but it is so much more intense and durable as the injection is colder, more abundant, and more frequently repeated.—*Medical and Surgical Reporter.*

